# Automated Workflow for Usability Audits in the PHR Realm

**DOI:** 10.3390/ijerph19158947

**Published:** 2022-07-22

**Authors:** José A. García-Berná, Raimel Sobrino-Duque, Juan M. Carrillo de Gea, Joaquín Nicolás, José L. Fernández-Alemán

**Affiliations:** Department of Computer Science and Systems, University of Murcia, 30100 Murcia, Spain; raimel.sobrino@um.es (R.S.-D.); jmcdg1@um.es (J.M.C.d.G.); jnr@um.es (J.N.); aleman@um.es (J.L.F.-A.)

**Keywords:** personal health records, usability assessment, computer-aided usability evaluation, usability heuristics, Method Evaluation Model, Technology Acceptance Model

## Abstract

Teleworking is an everyday reality in today’s world. Many companies are opting for this way of working remotely, as it improves employers’ satisfaction along with the work–life balance and it does not lead to any significant reduction in performance. In general, the comfortable use of software applications is a priority, and quality aspects such as usability are of vital importance. In this paper, a novel workflow is proposed. It consists of the following components: (1) a tool to manage usability evaluations based on expert reviews, called Usevalia; (2) a set of heuristics to be considered when performing the evaluations; (3) a catalogue of usability requirements to guide the evaluations; (4) a checklist related to the heuristics to verify that the evaluations have been performed correctly by all the auditors; and (5) a set of tasks to know in advance the functionality of the applications to be audited. Personal health records (PHRs) were taken as a case study. This methodology made it possible to involve a total of 31 working groups that evaluated the usability of four PHRs. Concerning the main results, the evaluators agreed that with Usevalia the usability evaluations could go a step further because they are coordinated and can work together remotely. In addition, analyses of usability scores provided insight into current proposals for improving usability of applications.

## 1. Introduction

Information systems (IS) are key in today’s modern societies [1]. They grow over the years and are the basis of most of the technological services enjoyed daily by millions of citizens. Software development companies are struggling to stand out in a very competitive market. They are increasingly numerous and generate a significant impact on the Gross Domestic Product (GDP) of countries [2]. The key to their commercial success lies in the development of quality software products that meet users’ needs, solve their problems, and are familiar to a large majority of people [3,4].

In many areas of everyday life ISs are present, such as healthcare, banking, e-commerce, etc. Between them, the health sector moves large budgets each year with estimates of over $100 million for the largest institutions [5]. Given the size and complexity of healthcare systems, it is essential that citizens make responsible use of them in order to achieve economic sustainability [6]. In this line, personal health records (PHRs) can enhance efficient healthcare use, as they allow patients to know at all times the evolution of the diseases they are suffering from. These systems store medical data that users can consult with an electronic device. Although they are not widely known by the majority, several studies indicate a remarkable willingness on the part of patients to share their data with the medical doctors [7]. These systems can be directly connected to hospital facilities, giving rise to tethered systems. They can also be found as standalone applications, both on cell phones and PCs, which connect to a server that provides access to the data. They are usually found on the web, and users can access their profile with an ID and password to update medical data. These systems can be free or paid. Between the PHRs in web format, Kaiser Permanente stands out with 12.5 million users served in 2022 (http://about.kaiserpermanente.org/who-we-are, accessed on 27 May 2022).

Convenience in the use of IS is key to meeting the functionality demands required by users [8]. In this vein, usability has been identified as one of the most important factors determining the success of software applications [9]. Correct design of software systems is a challenge for software companies to make their products widely known in the market. It is essential that usability aspects are taken into account during the testing of the applications in order to detect flaws to correct before launching the applications to the market.

Usability evaluations become relevant when developing software applications because they allow one to detect inconveniences or small annoyances that usually occur during the use of software applications, complicating their understanding and operation. It is worth mentioning that among all the software quality factors, usability enables users to use the systems in a comfortable way [10]. Therefore, usability is important to boost the adoption of software applications [9]. In particular, it is recommended that depending on the characteristics of the system to be analysed, the usability evaluation changes, allowing one to delve into specific aspects of the systems to be analysed [11,12]. The use of computer-aided usability evaluation (CAUE) tools to assist in usability evaluation is becoming a great help to gather more information and to involve a greater number of auditors [13]. This type of system allows one to centralise the usability analysis of software systems, and might provide auditors with usability requirements catalogues, instructions to proceed to evaluate usability, access credentials to the systems to be evaluated, and so on. With these tools the evaluations are clarified before to proceed, and more accurate results may be produced.

A usability evaluation workflow for PHRs was proposed in this paper. The core of the method is based on the centralisation of tasks, using for this purpose a CAUE tool developed by the authors called Usevalia. Moreover, the following components were added to the workflow:

1. heuristics adapted to agree on the concepts in usability to be evaluated by the auditors;
2. a set of tasks to learn how PHRs work; and
3. a set of usability requirements catalogues to propose a checklist, guiding the usability assessment process.

To better understand the strengths and weaknesses of the framework, the Method Evaluation Model (MEM) adapted to PHRs was applied. MEM [14] is a theoretical model for evaluating IS design methods. It incorporates two dimensions: actual effectiveness, and adoption in practice. Moreover, it combines Rescher’s theory of methodological pragmatism, which is a theory for validating methodological knowledge [15], and the Technology Acceptance Model (TAM) [16], which is a theoretical model for explaining and predicting user acceptance of IT.

To assess whether an IS will be used, MEM is appropriate and can be adapted to a particular system. In the case of PHRs, practitioners’ perception of effectiveness was considered much more important than adoption in practice. For this purpose, the effectiveness perception parameters measured the effects on the decision to use the framework [16,17,18]. Only perception effectiveness in MEM was previously hypothesised in the literature, and it was based on TAM and the Theory of Reasoned Action (TRA).

The beliefs that influence attitudes form the basis of TRA, which in turn lead to intentions and, finally, guide or generate behaviours. TAM adapts the relationship between belief, attitude, intention, and behaviour that arises with the use of IT to a model of user acceptance. The goal of TAM is to provide insights on the factors that determine technology acceptance. Moreover, it is capable of explaining user behaviour across a broad range of end-user computing technologies and user populations with brief and theoretical descriptions [16]. In a literature review conducted by Hu et al. [19], TAM was considered a valuable and useful model to explain and predict user acceptance of IT. This characteristic was remarkable for students in a university, and executives in a business organisation context.

The usability evaluations were carried out with the proposed workflow. As a result, it was possible to analyse the usability of PHRs, shedding light on the importance of employing adapted and well-defined methods in usability evaluations on domain-specific ISs. The following research questions were proposed to organise the research:

RQ1. What are the main Usevalia’s evaluation results for each variable of TAM?
RQ2. What are the results of the usability evaluations of the PHRs?

This paper presents a novel workflow for usability evaluation. To date, there is no other approach that addresses both theoretical and practical perspectives at the same time. The theoretical merits of the workflow are that it provides auditors with access to detailed information on how to proceed with evaluations. In particular, auditors can consult a catalogue of usability requirements, to get a precise idea of how usability should be implemented in systems; usability heuristics specifically designed for e-Health systems, which allow for a deeper understanding of the specific usability aspects to be assessed in the audit; and a checklist to reinforce the usability concepts of each heuristic. In this case, the checklist consists of a set of two questions for each heuristic. The practical merit of the workflow, which is the core of the proposed approach to usability evaluation, is the CAUE Usevalia tool. This tool forms the technological infrastructure provided for usability evaluations. It is available to auditors 24/7. It consists of an electronic information system that is used by the auditors as a database for training and documentation of the usability evaluations to be carried out. In this workflow, the auditors have all the necessary materials for performing usability evaluations in a remote location, which makes it possible to reach auditors all over the world and involve them with the simplification of the evaluation tasks.

The paper is organised as follows. Section 2 describes the PHR selection protocol along with the proposed workflow for assessing the usability of PHR systems. Section 3 presents the results of the parameters that highlight the benefits of using the CAUE Usevalia tool. In addition, the results of the usability evaluations of PHRs are shown. In Section 4, both assessments are analysed. In particular, Usevalia and the PHRs are evaluated for a better understanding of the results. Finally, Section 5 presents some concluding remarks and proposes future work.

## 2. Materials and Methods

This section shows the components of the experiment. First, the selection protocol of the PHRs is explained. Then, the heuristics used in the usability evaluations are described. A catalogue of usability requirements is presented, which served to reinforce the evaluation process. Based on this catalogue, the checklist, extracted from the literature, is reviewed. In addition, a set of common tasks in the PHRs is proposed to guide the evaluation. The Usevalia system is also described in this section. Finally, a MEM based on TAM is introduced, exposing the strengths and weaknesses of the whole workflow.

### 2.1. PHRs Search

The selection of the PHRs was based on a search protocol previously proposed in literature [20]. The search string “PHR providers OR PHR website” was used in the following databases to search for PHRs: Medline, ACM Digital Library, IEEE Digital Library, and ScienceDirect. The manuscripts found were read in order to detect PHRs. Reporting quality guidelines established by the Preferred Reporting Items for Systematic reviews and Meta-Analysis (PRISMA) group were taken into account to make the search as accurate and unbiased as possible [21]. The following inclusion criteria (IC) were taken into account: IC1, the PHR had to be free of charge and IC2, the PHR had to be available on the web. In total 19 PHRs were found.

A set of exclusion criteria (EC) were proposed in order to refine the search results. These were as follows: EC1, PHR not available; EC2, PHR not free; EC3, no login possible; EC4, PHR malfunctioning; EC5, PHR available only in the US; EC6, low popularity. In particular, EC6 could be applied by means of the Alexa tool (alexa.com/siteinfo, accessed on 27 May 2022), which allows us to rank web portals according to the number of daily visits. By observing the results obtained when assessing the popularity of web portals, a score threshold of 10 million was set to determine the low popularity of websites. This service was withdrawn on 1 May 2022 but the popularity analysis was carried out before that event.

**Figure 1:**
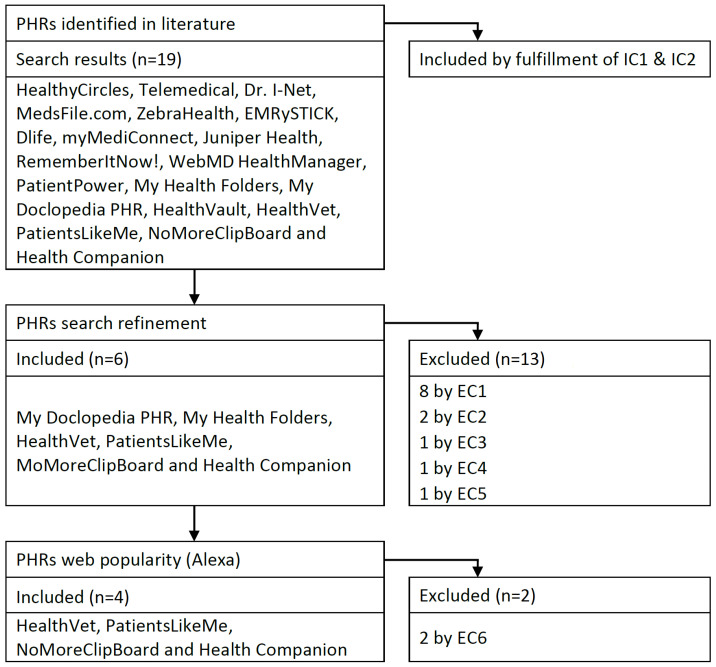
Research flow chart.

MedsFile, EMRySTICK, Dlife, Healthy-Circles, Dr. I-Net, Telemedical, ZebraHealth, and HealthVault were disregarded in the study by EC1 Juniper Health together with myMediConnect for meeting EC2, RememberItNow! was discarded by EC3, WebMD HealthManager was rejected by EC4, and PatientPower by EC5. When using EC6, My Health Folders and My Doclopedia were removed from the results. Figure 1 summarises the entire process and shows the selected PHRs.

### 2.2. Usability Heuristics

Usability evaluations of IS can be carried out by using different techniques such as cognitive walkthrough, think-aloud protocol, expert evaluation, etc. In this study, expert evaluation based on heuristics was employed [22].

Heuristics formed the basis of the evaluations. There is a wide variety of heuristics proposed in the literature. Of these, the 10 Principles of Design by Nielsen are well-known (please visit nngroup.com/articles/ten-usability-heuristics, accessed on 27 May 2022). They have been widely used in a vast number of studies. The heuristic can be adapted when evaluating particular ISs such as PHRs. In this study, a collection of heuristics based on the 10 Principles of Design by Nielsen, specially designed for eHealth systems, was employed [23]. They are as follows:

- Match: enable the graphic components in the user interface meets the mental model of users;
- Visibility: make visible that the system is processing;
- Memory: give information to the users on how to perform a task in the system;
- Minimalist: displayed only the basic information and avoid unnecessary elements in the GUI;
- Error: warn users from an error and provide information to solve it;
- Consistency: make easy to separate visually the different sections of the web page;
- Control: allow to undo or cancel any action taken in the system; and
- Flexibility: keep past actions on an historical log to allow reuse.

A checklist was also used [23], aiming at guaranteeing that the concepts considered in the evaluations between the auditors were the same. A total of two mapping questions were posed to this end. In addition, a usability software requirements catalogue was considered in this action. The catalogue in usability requirements was proposed by the authors of the paper and was validated in previous studies [24]. It is available at https://docentis.inf.um.es/catalogue/ (accessed on 27 May 2022) (user & password: requisite. It is required to filter the results to find the usability catalogue). The checklist is depicted in Table 1.

### 2.3. Tasks Performance

A collection of tasks were performed before to proceed with the usability evaluations. PHRs have particular functionalities in health, which had to be known by the auditors. To this end, the tasks were related with the common uses that can be done in these ISs. Moreover, a total of two user profiles, medical staff and patient, were taken into account to define with more accuracy the tasks in the evaluations [25,26]. The user profiles allowed us to simulate the mental model of the typical user when actions are taken in the PHRs. Moreover, recommendations from the American Health Information Management Association (AHIMA) were considered by the time of proposing the tasks [27]. These features provided a more complete evaluation of the PHR. Table 2 shows all the tasks carried out by the auditors before giving a score to each heuristics and go deep down in the assessments.

### 2.4. Usevalia Tool

Usevalia is an automated heuristic inspection-based tool for usability audits. It supports multi-users audits so that a team can work simultaneously on the same audit. The usability audit process in Usevalia starts when the chief auditor logs in with the username and password. Once logged in, any user has two options: (1) define the application to be audited, and (2) define the catalogue of usability guidelines to be used in the audit. When selecting the web application to be evaluated, the user must fill in a form, indicating the name, link address, web category, and description of the application. Next, define the scoring scheme that will be used to evaluate each of the usability guidelines. The scoring scheme can be created or deleted in the tool’s database. When creating a scoring scheme, each of the values is indicated (e.g., high, medium, low). The tool does not allow the auditors to delete a scoring scheme in the database. The teams of auditors that will participate in the usability audit are also managed within the same tool by the chief auditor. Thereby, when creating an audit team, the name of the team and the users who will play the audit role must be selected together with the team description. An audit group cannot be deleted if it has been associated with an audit until the audit is closed.

**Figure 2:**
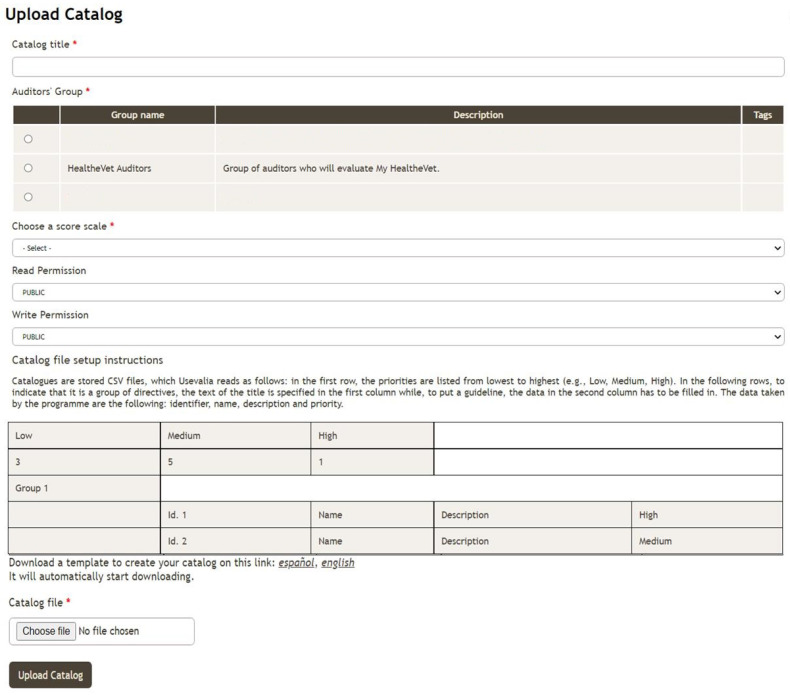
Usevalia’s upload catalogue functionality. The * indicates that the data is mandatory.

The chief auditor can import a catalogue that will be used to assess the system under test in a new audit. A repository of templates can be accessed from the Upload/Download catalogue page to create a collection of guidelines or to reuse a previously defined catalogue (see Figure 2). The catalogues are stored in CSV files, which Usevalia reads as follows. In the first row, the priorities are listed from lowest to highest. In the following rows can be found the items of the catalogue. In the first column appears the title. Moreover, the guidelines of the catalogue are indicated in the second column. For each guideline, the following information is stored: identifier, name, description, and priority. In the form, the catalogue’s title, the team to which it will belong, the scoring scheme, and the read/write permissions must be filled in before uploading the catalogue. The chief auditor is the only one who can delete the catalogue from the application when it is not being used in another audit. A catalogue employed in the usability evaluations of the PHRs can be downloaded in CSV format at http://umubox.um.es/index.php/s/IDWChOi6jy6uAyS (accessed on 27 May 2022).

Subsequently, the chief auditor defines the type of audit (e.g., basic, standard, task-based). Next, the auditor or team of auditors can start to evaluate the application by using the pre-loaded guideline catalogue. Each auditor then performs the heuristic evaluation. If there are x days left before the end of the audit, the system sends a reminder e-mail to the auditors performing the assessment so that they are aware that the time limit is running out. Once the audit expiration time is over, the audit is automatically closed.

Finally, Usevalia allows one to create, delete, evaluate, close, reopen, generate, and display the statistics of an audit. Some of the statistics displayed are: the total percentage of passed and failed guidelines in the audit, the number of auditors who have indicated a guideline as passed/failed, and the number of passed/failed guidelines at each priority level. Once the lead auditor has selected an audit to generate a report, they will receive a link to download the report. The file will be in PDF format and will contain all information related to the selected audit. The tool is available to the HCI community at the following address https://giis.inf.um.es:446/drupal2/usevalia (accessed on 27 May 2022). It is worth noting that the Usevalia interface was first structured following the authors’ experience on web design, and secondly the usability of this interface was evaluated by means of the authors’ usability evaluation workflow, which provided some tips to improve the final design. In terms of user experience, Usevalia is a user-friendly system. This application is an aid to auditors in usability evaluations, as they can find all the information they need to carry out the audits. The system interface is simple and intuitive. In Usevalia, all options are displayed in a column on the left side at all times. Specifically, the menu displayed in the left-hand column consists of the following sections: Apps, Auditor Groups, Scoring Scales, Catalogues and Audits. In the first option, Apps, it is possible to create, edit or delete a record of an application to be assessed. Basic information about the app is stored in each record: name, category, URL, and a brief description. The second option stores the participants who will perform the audits. This information is editable at any time. In the next option it is possible to create different scales to score the heuristics considered in each usability evaluation. To guide the usability, it is possible to consult usability catalogues in the following option along with the checklist. Finally, the Audits option allows us to manage the outcome of the audit work. Figure 2 shows an example of the look and feel of Usevalia.

### 2.5. Usability Assessment Protocol

**Figure 3:**
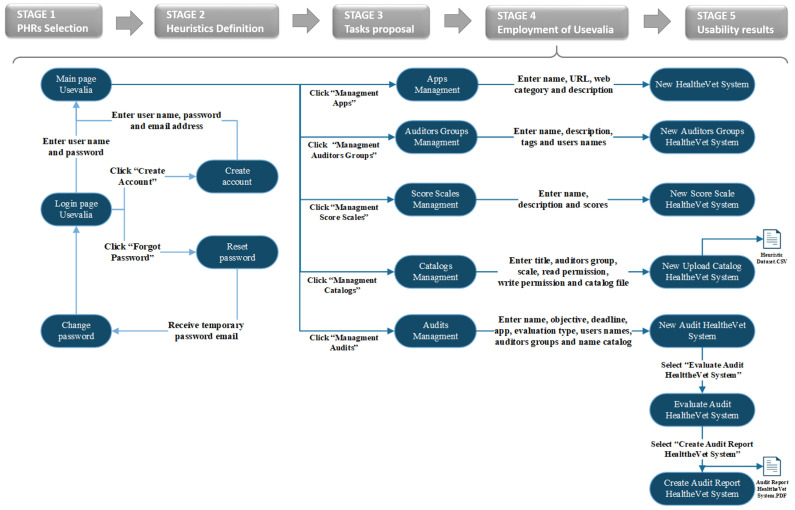
Workflow followed in the usability assessments.

Usability evaluation is a complex task. A protocol has to be established so that all auditors perform an evaluation under the same conditions [28]. The whole evaluation protocol consisted of the following items: (1) Usevalia tool, (2) adapted heuristics, (3) performance of the proposed tasks and, finally, (4) the evaluation of the PHRs based on the heuristics and the checklist, keeping the usability requirements catalogue available for the auditors. All this information was held in Usevalia, and the auditors could consult it at any time in any place with an electronic device. Figure 3 shows the whole workflow, with an audit process of HealthVet using the CAUE Usevalia tool.

In this study, the audits were conducted by experts in usability employing Usevalia. The experts had experience in user interface design and software quality factors such as accessibility and usability. In particular, the work was carried out by students of the User Interfaces course, which is a subject taught in the first semester of the fourth year of the Degree in Computer Science at the University of Murcia. This subject deals with aspects of the design of graphical user interfaces from the perspective of the software quality. In groups of two, students chose the PHR to be evaluated between the ones presented in Section 2.1. The first step carried out in the evaluations was to perform all the tasks proposed in Table 2. After a thorough examination of the PHR, each one of the heuristics was scored on a Likert scale ranging from 1 (i.e., very little supported) to 5 (i.e., very well supported). A value of 0 meant not supported.

### 2.6. Method Evaluation Model (MEM)

In this paper, a total of 7 variables were defined to assess the experience of employing Usevalia. These variables were determined using MEM [17], which was adapted to assess the skills and knowledge of auditors to perform usability audits with the CAUE tool. The variables were classified into 4 types: (1) performance-based variables, which measure how well subjects can use a tool to conduct usability audits in the PHR realm (i.e., effectiveness and productivity); (2) perception-based variables, which measure the perceived usefulness (PU), perceived ease of use (PEU), and perceived attitude (PA) that subjects believe a usability audit tool has; (3) intention-based variable, which measure the perceived intention of auditors to conduct audits with Usevalia (i.e., intention to use), and (4) behaviour-based variable, which measures the actual usage of Usevalia in the PHR audits (i.e., actual usage).

**Figure 4:**
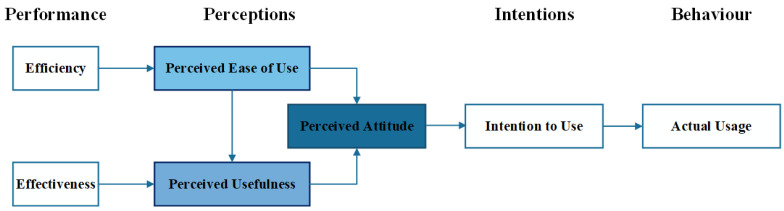
MEM adapted to the audit of PHR systems based on Usevalia.

Figure 4 depicts MEM adapted to the audit of PHRs based on Usevalia and TAM, which are both employed in the study. It is a reduced TAM, excluding behaviour-based and performance-based variables. In this context, the behaviour-based variable (i.e., actual usage) is not evaluated because, on the research side, several prior empirical studies have reported a strong, significant causal link between intention to use and actual behaviour [17,19,29,30]. It is thought that the degree to which Usevalia was easy to use, as perceived by auditors, affected both their perception of the usefulness of the tool and their attitude toward using the tool in general. Attitude is also influenced by the level of the tool’s usefulness, as perceived by the auditors.

The TAM questionnaire on the use of Usevalia was filled in by a total of 22 students of the user interfaces course who carried out the usability evaluations. All these students shared a common background concerning user interface evaluation. This questionnaire contained a total of 18 questions and was divided into 3 blocks. They were the following ones: Block 1: PU; Block 2: PEU; and Block 3: PA. Block 1 was composed of questions Q1–Q12, Block 2 consisted of questions Q13–Q16, and Block 3 encompassed questions Q17–Q18 (see Table 3).

## 3. Results

This section presents the results of the evaluation of the benefits of using the Usevalia tool together with the usability assessments of the PHRs. Subsections relating to the research questions have been created for a better structuring of the information.

### 3.1. RQ1. What Are the Main Usevalia’s Evaluation Results for Each Variable of TAM?

The goal was to employ TAM to investigate user’s behaviour by means of the PEU, PU, and PA variables.

#### 3.1.1. Perceived Usefulness (PU)

Looking at the results obtained for PU, it can be said that, in general, students perceive Usevalia as a useful tool for conducting usability audits. For example, 72.72% thought that the use of Usevalia would improve students’ performance in human–computer interaction (HCI) (Q1). Only 4.54% of the respondents think that it would not help them in the teaching–learning process of HCI subjects (Q2). Along the same lines, 63.63% consider Usevalia to be a step forward in carrying out usability audits (Q3), while 59.09% agree that it is better than Microsoft Excel™ for carrying out usability audits, specifically audits with heuristics evaluation (Q12). Also, 45.45% agreed that the tool handles users and user roles well enough to be used in a real project (Q5), and 27.27% indicated that they disagreed with this aspect.

**Figure 5:**
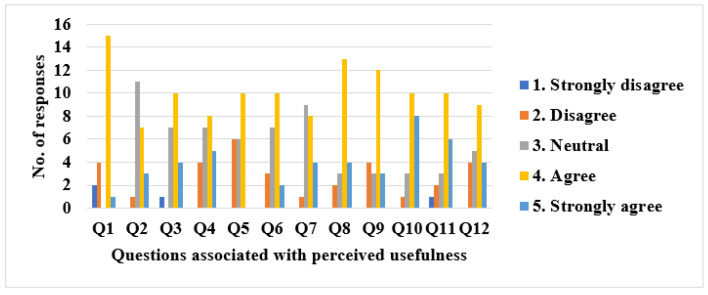
Number of responses for items related to PU.

Regarding the implemented functionalities, 54.54% agreed that the included web categories (Q6) and the different types of predefined tasks associated with the web categories (Q7) seems to be sufficient to represent all web pages/applications. A total of 77.27% of students consider that the different types of evaluation (Basic, Standard, Task-based) of the tool seem to be sufficient to evaluate an audit (Q8). Also, 81.81% agreed that the reports generated by the tool helped to understand the final results of an audit (Q10), whereas only 13.63% did not agree that the Usevalia graphs were useful to interpret the audit results (Q11). Finally, 68.18% considered that Usevalia streamlines the audit assessment process (Q9). Figure 5 shows number of responses for the items related to PU.

#### 3.1.2. Perceived Ease of Use (PEU)

**Figure 6:**
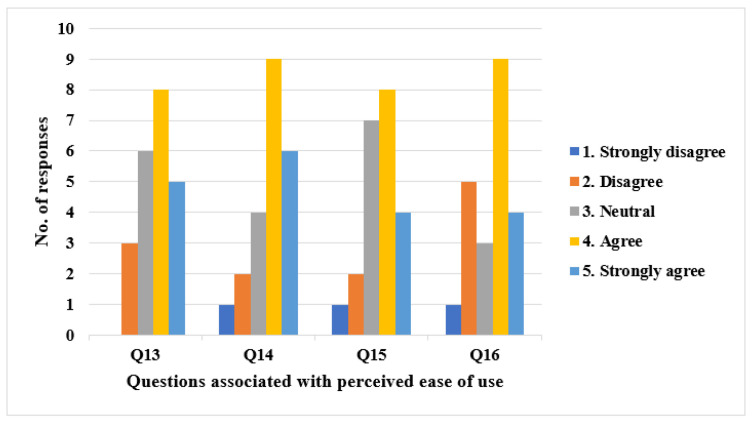
Number of responses for items related to PEU.

Concerning the PEU, the analysed data indicate that 59.09% of respondents consider that learning how to use Usevalia is easy (Q13). On the other hand, 68.18% find the interaction with this tool to be clear and understandable (Q14). Another aspect was to assess the intuitiveness of the steps to follow in the tool before creating an audit (Q15). In this case, 54.54% of respondents agree or strongly agree with this statement, whereas the percentage of respondents who disagree or disagree respectively is 13.63%. Finally, 59.09% agreed or strongly agreed that they had no problems in using and understanding the way in which Usevalia works (Q16). Figure 6 depicts number of responses for the items related to PEU.

#### 3.1.3. Perceived Attitude (PA)

**Figure 7:**
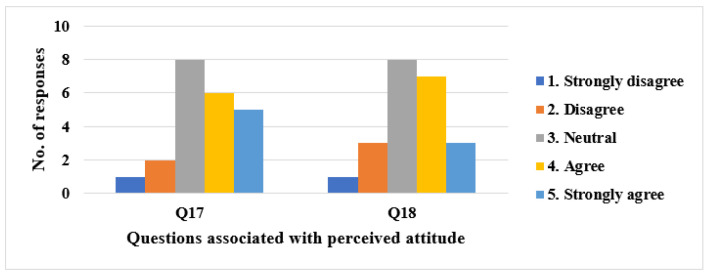
Number of responses for items related to PA.

Regarding the PA, half of the respondents would use Usevalia to conduct usability audits (Q17). Moreover, 45.45% would use it to improve their performance in the HCI course, whereas only 18.18% consider that they would not (Q18). Figure 7 shows the number of responses for the items related to PA.

#### 3.1.4. Correlations

The correlation analyses have shown significant relationships between the variables included in TAM (see Table 4). The values obtained for the correlations ranged from r = 0.546 to r = 0.853. The highest correlation coefficients were found between PA and PU (r = 0.853; *p* = 0.000), followed by the relationship between PEU and PU (r = 0.637; *p* = 0.001), and finally between PA and PEU (r = 0.546; *p* = 0.009).

### 3.2. RQ2. What Are the Results of the Usability Evaluations of the PHRs?

A total of 7 groups of auditors participated in the usability evaluation of the HealthVet portal, 4 groups analysed the NoMoreClipBoard portal, 15 groups studied the usability of PatientsLikeMe and 5 groups were in charge of evaluating Health Companion. The auditors were randomly grouped in groups of two. No particular characteristics were taken into account in the auditors’ associations. The scores given to each heuristic were averaged with the tasks, providing an estimate of the level of implementation of the heuristic in the PHRs. These average values are shown in Table 5, Table 6, Table 7, Table 8 and Table 9. Moreover, all the results of the usability evaluations can be found at http://umubox.um.es/index.php/s/IDWChOi6jy6uAyS (accessed on 27 May 2022), in the UsabilityEvaluations.xlsx file.

In this study, a score greater than or equal to 3.5 out of 5 was taken as a considerable level of usability [31,32]. The average of all scores given by auditors across all the PHRs was 3.43. Usability assessments with values higher than 3.5 were analysed in detail in Section 4. Based on the results, it can be observed that the Match stood out in all the PHRs. A high score in this heuristic indicates that the terminology used in these systems is similar to the one that can be used in the real world, avoiding confusion when selecting the actions to be carried out. The Visibility heuristic was highlighted in the PHRs NoMoreClipBoard and PatientsLikeMe. The fact that these PHRs received a high score on this heuristic indicated that both systems are very visual, and they display enough information for a more comfortable use of the system. The heuristic Memory stood out in the PHRs HealthVet, NoMoreClipBoard, and Health Companion. This result shows that the PHRs were more convenient to use, as there was sufficient information at all times to know how to perform the tasks in the system. The Minimalist heuristic stood out in the PHRs PatientsLikeMe and Health Companion. This result shows that in these systems only the information needed to perform the tasks was displayed in a summarised form. The Error heuristic scored one of the lowest values. This shows that the system did not indicate sufficiently in advance and with enough information when a PHR error was going to occur. In HealthVet, NoMoreClipBoard, and Health Companion, the Consistency heuristic stood out. This indicated that it was generally easy to find the information sought in the system. In HealthVet and Health Companion, the Control heuristic stood out, which is related to the possibility of being able to do and undo actions in the systems. Finally, the Flexibility heuristic stood out in Health Companion. This result tells that the system offers several alternatives to perform the same task.

## 4. Discussion

The results shown above were evaluated to draw the main findings of the study, dividing the section according to the research questions. In particular, how the use of the Usevalia tool influenced the usability evaluations was evaluated. In addition, an analysis of the results obtained in the usability evaluations was carried out.

### 4.1. RQ1. What Are the Main Usevalia’s Evaluation Results for Each Variable of TAM?

The present study empirically validates TAM from the perspective of using an audit system to assess the usability of PHR systems, investigating the difference between conducting an audit with an automated tool, and manual audits with a checklist. The results of this study offer significant implications for the acceptance of Usevalia both in academia and in practice. Based on the results of the evaluation of TAM variables, this study positively addresses the data collected, the factors, and the users’ intention to use Usevalia for usability audits.

Several studies agree on the positive and significant relationship between TAM variables (PEU, PA and PU) [33,34,35]. The two most important variables in predicting the development of a technological tool with TAM are PU and PEU [36,37], which have a direct relationship with the variable Intention to Use [38]. The PU assesses the degree to which respondents believe that the use of Usevalia can improve the usability audit process [34], which is an important aspect to evaluate as it has been shown to have a direct influence on the use of the tool by users [39]. According to the results, Usevalia was considered a step forward (Q3). However, there is a large percentage of respondents who in many cases were neutral about whether or not Usevalia improves the usability audit process (31.81%).

The second variable considered crucial is the PEU, which evaluates the degree to which respondents consider that Usevalia will not require any extra effort to use it [34]. The results can be considered very positive, as all questions related to this aspect received more than 60% favourable responses. The results indicate that users had no difficulties in handling the Usevalia CAUE tool. Because the respondents are familiar with certain types of social software, in this case, the good results obtained in this respect can be associated with the fact that it is a simple tool to use.

The PEU variable showed a significant influence on users’ intention to employ a system in a specific context, such as online banking services [40]. In the context of conducting usability audits, a system to support the audit process such as the CAUE tool Usevalia is used by individuals for specific purposes. Therefore, individuals are primarily concerned with whether or not the services offered by the system are useful to improve and simplify the audit process. If individuals perceive that, although the system is easy to use, it does not improve their auditing activities, their attitude towards the use of Usevalia will not improve in any way.

Another important aspect to take into account when assessing the acceptance of a tool is the extent to which users are interested in using it. This is done by analysing the attitude variable, which indicates the user’s willingness to employ the tool [16]. It can be said that the users’ attitude is quite favourable. In all the questions related to this variable, the answers of the respondents have been quite positive, since in all cases the majority have agreed or strongly agreed. Usevalia would be used by respondents if they needed to perform usability audits. On the other hand, the percentage who disagreed or strongly disagreed was low (15%).

The correlation analyses were carried out to test the relationships between the different variables included in the model. The results showed that all the variables were significantly and positively related, which validates the idea that these factors are of vital importance in the acceptance or use of a given system [41,42].

### 4.2. RQ2. What Are the Results of the Usability Evaluations of the PHRs?

This section has been subdivided by PHRs. For each PHR, heuristics with a score greater than or equal to 3.5 were studied. This threshold was set to find out which heuristics stood out from the rest. The results of the usability evaluation of the eHealth portals were used to highlight features of the GUIs that improve usability.

#### 4.2.1. HealthVet

HealthVet offers support to ex-military personnel during the recovery process for the after-effects they suffer from. An average score of more than 3.5 was obtained in half of the heuristics. The heuristics were as follows: Match (3.96), Memory (3.67), Consistency (3.88), and Control (3.81).

#### Match (3.96)

HealthVet is a health system developed by the US government, which is aimed at former exercise veterans. It has a strong government iconographic presence which brings officialdom to the web portal. This feature enhances the Match heuristic as it makes the web portal familiar to military members who have routinely looked at the iconography in the past [43,44]. In addition, it has two types of account, basic and premium. The basic account allows any user to upload medical information to the portal. The premium account offers greater functionality, allowing one to view electronic medical history registered with the US Department of Veterans Affairs (VA), send secure messages to the VA medical team, manage VA appointments, and download health summaries. The use of this type of account is restricted to members of the US military, which distinguishes them from other users in a similar way to premium services in the real world. In addition to being distinctive, premium products have been shown to attract user attention [45]. Concerning quality assessment studies of premium software, it was observed that this type of application has better quality attributes [46]. Various methodologies have been employed to design premium software. In one of them, all stakeholder viewpoints were collected in a single structural view, showing the central decision in the architectural design [47]. In another, a new customer learning framework based on synergies between blocks of both direct and indirect learning processes is proposed to design freemium and premium versions of the software [48]. The development of software with premium features should be put in place to improve quality.

#### Memory (3.67)

**Figure 8:**
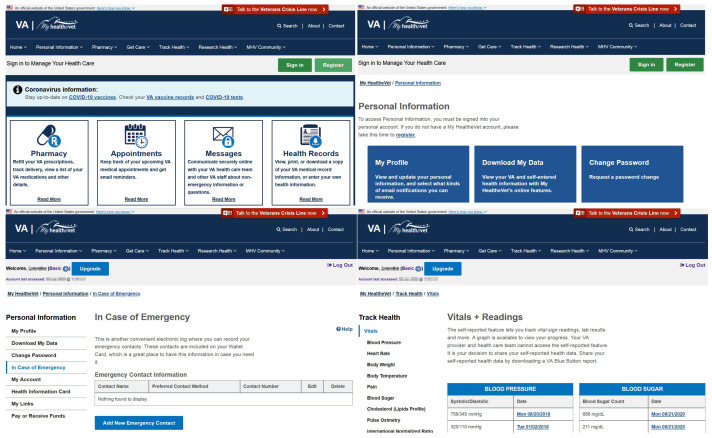
HealthVet screen captures.

The Memory heuristic also stood out in this PHR. In HealthVet, it is very common to find comments indicating what can be performed in each of the widgets. This information allows speeding up the tasks to be performed in the PHR, because they reduce the search time for information of interest. This type of information is important in GUI design. In literature, Offer Informative Feedback has been identified as one of the most cited GUI design principles [49]. In particular, the messages displayed in HealthVet are descriptive and can be found without logging into the portal. This feature claims to use the system. Furthermore, the short messages guarantee that they can be read, which avoids the well-known tl;dr (i.e., too long; didn’t read) [50]. Some examples of small explanatory sentences can be found in Figure 8.

#### Consistency (3.88)

**Figure 9:**
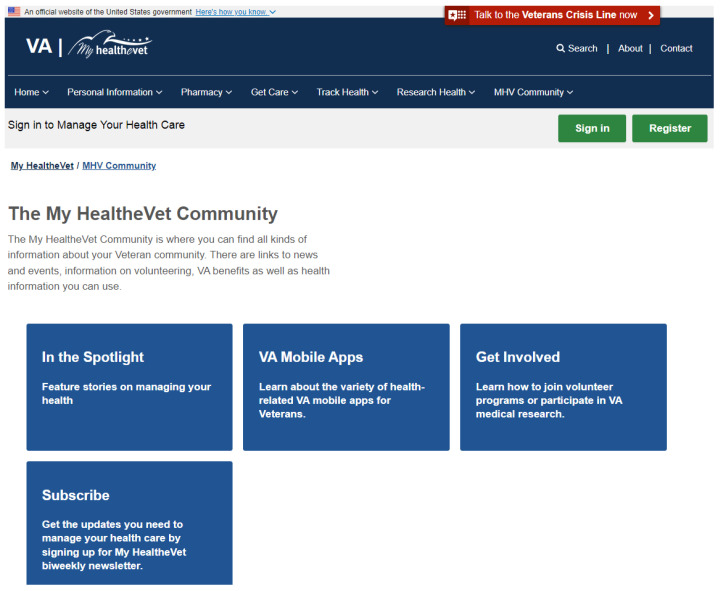
My community section in HealthVet.

The Consistency heuristic also featured prominently in this PHR. In HealthVet, users are encouraged to use the web services. In addition, social awareness is promoted, aiming to generate a sense of community with calls to action for participation in activities and sign up in the web portal. This information is separated in HealthVet with a section named My community (See Figure 9), which brings Consistency to the portal. This section aims at giving support to ex-military personnel in their medical situations. It allows one to know about stories from the members, download mobile applications for healthcare, participate in volunteer programs, and give access to a newsletter. All the information is presented with simple and easy-to-understand nomenclature.

An example of the usefulness of appropriate information dissemination could be the case of mental health treatment, which is of vital importance, especially in critical situations such as the current COVID-19 pandemic. Patients who already have some kind of mental pathology suffer a greater impact in this exceptional situation [51], particularly in anxiety, depression, stress, and distress [52]. Moreover, there is a prevalence of major depressive disorder of around 45% [53]. In the literature, supports for coping with mental health were grouped into 4 categories: (1) informational (training, guidelines, prevention programs); (2) instrumental (personal protective equipment, protection protocols); (3) organisational (staff allocation, work schedules, reorganisation of facilities/structures, provision of rest areas); and (4) emotional and psychological (psychoeducation and training, mental health support team, peer support and counseling, therapy, digital platforms and telecare). This categorisation of mental support sought to generate interventions and guidelines that would enable governments to develop evidence-based policies to prevent or reduce the immediate and long-term impact of critical mental health conditions [54]. HealthVet covers each of the 4 categories mentioned above, impacting on the usability of the system as well.

#### Control (3.81)

HealthVet allows one to delete all health records entered manually in the Track Health section. It could not be ascertained in this study whether this functionality is available for premium users. In addition, there is also a HealthVet switchboard phone number at the bottom of the page for any issues regarding the use of the PHR. Customer service is a common service that is necessary in order to provide confidence to the customer when deciding to subscribe to a service or product [55]. HealthVet links to a telephone number for veterans in crisis, called the Veterans Crisis Line (http://www.veteranscrisisline.net/, accessed on 27 May 2022) and visible in both the header and footer. This emergency service has been studied in literature and has proven to be effective and successful in critical situations [56,57]. The availability on the part of the VA is undeniable with the presence of these forms of communication. In addition to telephone numbers, there is also the possibility of communicating by SMS, confidential chat, and teletypewriter (TTY). The diversity of ways to contact the customer in HealthVet was particularly varied. This characteristic empowers the users to employ the service offered in the web portal [55,58]. It has been shown that the fact that users have control over the information stored on web portals improves usability, increasing the sense of security of personal information displayed on the IS. This is an important feature in a PHR aimed at ex-military personnel [59].

#### 4.2.2. NoMoreClipBoard

As in the previous PHR, in NoMoreClipBoard half of the heuristics obtained an average score higher than 3.5. These were Match (3.91), Visibility (3.52), Memory (3.60), and Consistency (3.60).

#### Match (3.91)

**Figure 10:**
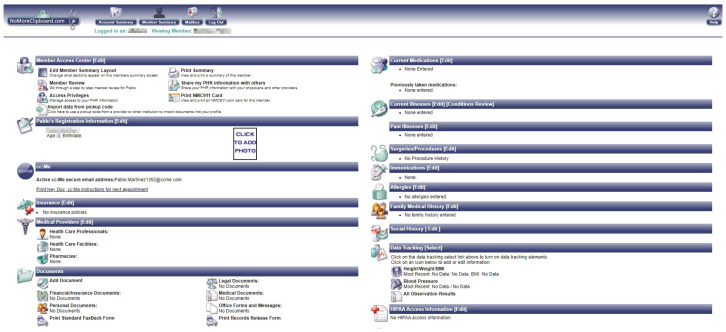
NoMoreClipBoard screen capture.

NoMoreClipBoard is aimed at all audiences. This PHR stood out from the rest by displaying all medical information when entering the user profile. This feature can generate cognitive overload [60]. To address this drawback, the system organises medical information into thematic icons, making it easy to quickly locate the information of interest. It has been shown in literature that a strong presence of thematic icons in web portals facilitates locating system options without creating misconceptions [61]. Essential usability concepts have also been studied concerning thematic icons. In this regard, user-centred interface design approaches were carried out in the literature. As a result, (1) user cognitive load, (2) effectiveness, (3) usage efficiency, and (4) user impressions were usability concepts that stood out from the rest [62]. The use of thematic icons drawn from the real world has a direct impact on these 4 usability factors. Therefore, in situations of information overload, it is advisable to have ways of organising the information displayed on the screen that build confidence in the use of the system [63]. Figure 10 shows the icons present in NoMoreClipBoard. As an example, in the documents section, a folder is shown; in the medications section, a pill logo is depicted; in the current illnesses section, a thermometer and a hot-water bottle are displayed. This way of presenting information was unique to NoMoreClipBord. By using these icons, real-world concepts are expressed and users quickly benefit from their association.

#### Visibility (3.52)

NoMoreClipBoard is a system with a strong presence of descriptive icons. It stands out for offering mechanisms that make it easier to find clickable links. These are a change of colour in the widget, a change of the mouse pointer icon and underline or not the hyperlink. These ways of identifying clickable links on web portals is well known. However, it may result in a cumbersome use of the web page when it presents an overload of widgets [64,65]. In fact, it has been found that the use of technology can lead to digital overload, which can negatively affect user exhaustion and performance [66]. Visual attention in a viewing area varies depending on the device used. On mobile devices visual attention is driven by a combination of bottom-up and top-down processes. However on a computer screen, visual attention follows a primarily top-down process. Highlighting web page links with a certain colour helps to attract attention and increases the users’ sense of control when performing a task in the system. Because searching for links on web pages is likely to be done from the top left to the bottom right, research suggests that a link to a website that is in the top left corner or displayed in a different colour in the middle area will attract users’ attention and provide a more effective user experience [67]. The aforementioned features can be found especially in the user profile of NoMoreClipBoard, boosting the Visibility heuristic.

#### Memory (3.60)

The Memory heuristic is enhanced in this PHR with informative messages. These messages indicate what can be done in the widgets. In particular, the messages displayed in NoMoreClipBoard are less abundant and more schematic than those found in HealthVet (see Figure 10). In any case, this favours rapid reading of the messages. It is important to note that the format of the messages is adapted to the use that will be made of them, so that they do not go unnoticed [68,69]. NoMoreClipBoard has a feature called Member Review. This feature allows for the display of a compilation of all the medical information sections in the PHR. In addition, it allows one to take a guided tour in order to check and complete the health data. During the Member Review tour, informative messages with brief instructions are displayed.

Exposing informative messages does not always lead to a better user experience of the PHRs. It has been observed that feedback is not always well received in decision making, and can even generate discomfort. This situation has a particular impact on clinical staff, who do not always welcome recommendations on their diagnostic performance. Therefore, it is advisable to analyse the way in which feedback is presented to the users of a system [70,71,72]. In this sense, the disabling of informative messages could be welcomed by experienced users of the PHR. NoMoreClipBoard is not aimed at any particular target audience. It is not intended to be a diagnostic tool, far from it. The messages found in this system are simple and general, making the Memory heuristic gain relevance for an acceptable level of usability of the PHR.

#### Consistency (3.60)

**Figure 11:**
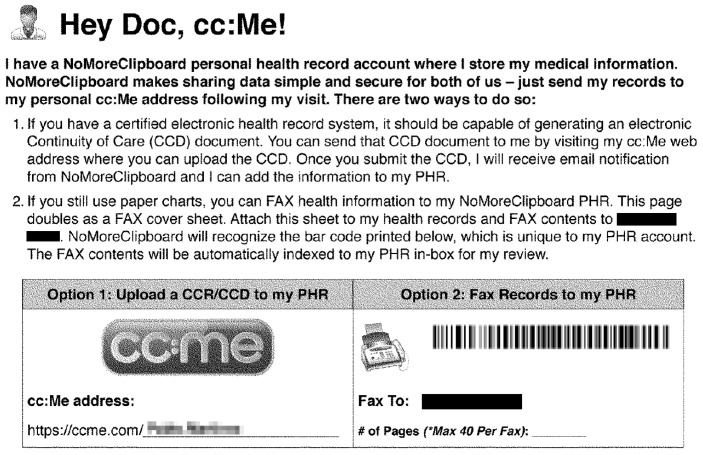
Cc:me feature in NoMoreClipBoard.

NoMoreClipBoard has a mechanism to speed up data loading, avoiding manual data entry. This feature is called cc:me. Its name is related to the concept of sending e-mails to copied senders. Each user has a cc:me address or a unique barcode that can be shown to the physician to dump medical information into the user’s profile. Naming this utility this way emphasises the Consistency heuristic, because the cc:me widget is associated with sending the medical data to the patient. This characteristic allows patients to have access to the information and prevents it from being stored in the facility, giving access only to physicians. Figure 11 shows the cc:me card downloadable in the user profile.

There must be alternatives to automate the entry of medical data into PHRs. This would save time for users, who would only have to check that the data entered are correct. The Lazy User Model has been used to demonstrate that when physicians manage consumers’ health information, it is the preferred solution, because it requires the least effort from patients and physicians [73]. On the other hand, this type of technological proposals have to be accompanied by mechanisms for storing and sending health data, ensuring maintenance and quality control [74]. Various mechanisms have been proposed in the literature to facilitate access to health information from multiple sources. To this end, blockchain smart contracts can be developed to manage access to medical information by patients, physicians, and healthcare providers [75]. In addition, an access control model based on the credibility of the requesting user can be implemented. This mechanism quantifies user trust based on historical visit records in the system, and the trend of historical user behaviour with regression analysis models. The results showed that the accuracy of trust and prediction of trust trends was better than with existing models [73].

#### 4.2.3. PatientsLikeMe

In a total of 3 heuristics, the mean score was higher than 3.5 in PatientsLikeMe. The heuristics were: Match (3.63), Visibility (3.55), and Minimalist (3.51).

#### Match (3.63)

**Figure 12:**
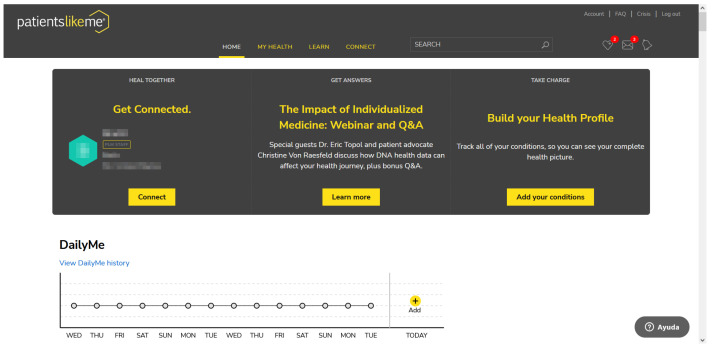
PatientsLikeMe Home.

In the header of the PatientsLikeMe Home there is a widget that enhances the concept of logical order of information stated in the Match heuristic (see Figure 12). This widget shows a set of text boxes that organise the information in a certain way. First, there is the Heal Together, Get Connected text box. In addition, there is a small piece of information from one of the PatientsLikeMe Staff Members. The Staff Members provide medical news in the PHR, motivating users to maintain a healthy lifestyle. Within the aforementioned section, it is possible to click on the Connect link. The purpose of this button is to access the profile of the Staff Member and communicate any remark. Subsequently, there is the Get Answers text box, which displays messages inviting users to learn more about the most common medical conditions. This section links to a small medical library with easy-to-read and brief information. Finally, there is a text box called Take Charge with which the attention of users is attempted to be captured for building the medical profile.

Individuals’ reasoning suggests that it comes from thinking beyond premises and using prior knowledge. It has been analysed how divergent thinking can be a predictor of logical reasoning. As a result, originality, in addition to fluency and cognitive ability, was a predictor of logical reasoning [76]. Along these lines, many researchers are beginning to consider the possibility of combining external knowledge in dialogue systems by using neural networks to generate informative and context-specific responses [77]. Endowing these systems with divergent thinking could facilitate the use of dynamic widgets that change the information in a logical way in order to capture the attention of the users. The aforementioned sections could be adapted to the context of each user in order to promote self-care.

#### Visibility (3.55)

**Figure 13:**
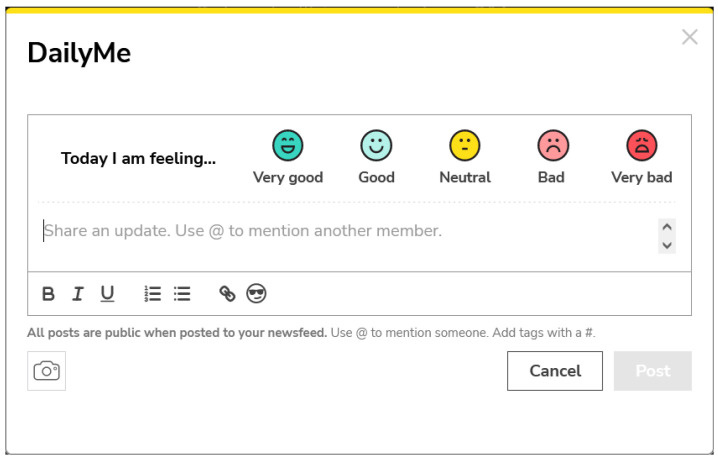
DailyMe feature.

PatientsLikeMe has a particular mood query functionality for users. On a daily basis, members can answer a short question in which different moods are represented with a Likert scale. This scale uses icons ranging from very bad to very good (see Figure 13). The results are stored in the PHR, showing the answers of the last 2 weeks in a graph of the Home. The objectives of this widget are multiple. The fact that it is displayed as soon as one enters the PHR gives importance to the widget. It has been shown that maintaining a good mood allows one to face a medical situation in better conditions [78]. In addition, there is a higher success rate in medical treatments when faced with a positive attitude [79]. The user by viewing the mood history can be aware of the internal situation and try to improve it if a negative mood history is accumulated. In this vein, humans are more likely to try to adapt their mood towards positivity when they receive positive messages [80,81]. A positive history of mood states could help to maintain patients’ good mood. On the other hand, this feature allows the discovery of aspects of daily life in users that promote positivity, which can be exploited to feed back good moods [82,83]. There are many therapies that have proven to be effective in favour of positivity such as laughter therapy, music therapy, etc. [84]. PatientsLikeMe could add simple widgets based on these types of therapies that would enhance the good mood of patients. In summary, displaying the information visually highlighted with mood icons and history allow to address a feature, such as mood, that medical professionals cannot address except with medication.

#### Minimalist (3.51)

Generally speaking, PatientsLikeMe is a PHR that presents a minimalist design. The use of the yellow colour facilitates minimalism, since yellow is one of the colours most easily captured by the human eye [85]. The colour palette selected in PatientsLikeMe makes it easier reading the text and locating the icons, boosting HCI. How the colours of widgets impacts the efficiency of visual search has been studied [86]. Participants in one experiment found GUI objects more quickly when medium luminance contrast was used rather than low- or high-luminance contrast [87]. By using mainly white shades, this quality is more likely to be fulfilled and make the search for information in the PHR more effective. In this way, minimalism has a positive impact on usability. Another aspect to note in the simplicity of the PatientsLikeMe’s GUI is that the widgets are widely separated from each other. This feature provides convenience when using the PHR. However, the icons displayed are 2D (flat icons). It has been observed that the use of 3D icons (pneumorphic icons) better captures the attention of users [88].

#### 4.2.4. Health Companion

Health Companion was the PHR in which the most heuristics obtained a mean score higher than 3.5. This result may tentatively lead one to think that it is the PHR with the best usability of all those analysed. In total, there were 6 heuristics with a score higher than 3.5, and they were as follows: Match (4.17), Memory (4.19), Minimalist (4.26), Consistency (4.25), Control (3.82), and Flexibility (3.83).

#### Match (4.17)

**Figure 14:**
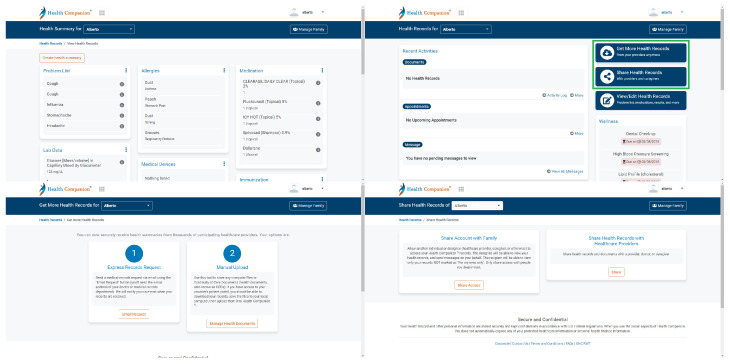
Health Companion screen captures.

Health Companion is characterised by displaying health information grouped in blocks (see Figure 14). Each of these blocks has a highlighted title to quickly find the information of interest. Clicking on the title opens the box and displays more information. This way of organising information reduces the cognitive load [89]. In addition, it allows information to be depicted in a thematic order, which has an impact on the Match heuristic. It has been studied how users perceive the information represented on web pages in terms of visual aesthetics. In this regard, visual complexity is one of the prominent aesthetic characteristics. As an example, the results showed an impact on the preference for using web pages depending on this aspect. In addition, a moderating effect on customers’ purchase interest in e-commerce portals was observed depending on the complexity of the web page [90].

#### Memory (4.19)

Upon entering the PHR, a set of buttons related to health records appears on the right (highlighted in Figure 14). The first widget is called Get More Health Records and offers two alternatives: Express Records Request and Manual Upload. In the former, health data can be requested from the physician by sending an email from Health Companion. The PHR itself has an e-mail client embedded in the system to send and receive e-mails from physicians. In the latter, documents with health data can be uploaded to the system. The next widget, Share Health Records, offers two alternatives: Share Account with Family and Share Health Records with Healthcare Providers. Users can create profiles for family members and configure on this page the access they give family members to their medical data. Another option is to allow physicians to access the medical data documents mentioned above. For this, the caregiver’s e-mail address is required. In the widgets mentioned, Get More Health Records and Share Health Records, detailed information appears to explain what each of the options in each widget consists of (see Figure 14). These explanations prevent errors when managing medical data in the PHR.

There must be an appropriate balance between the number of widgets and the design of the web page. In the literature, the aesthetics of a large set of web pages were analysed. The results showed significant differences between aesthetic and non-aesthetic web pages, taking into account aspects such as the position and average number of widgets [91]. In addition, deep learning was used to automatically calculate and quantify the aesthetics of web pages [92]. For this purpose, user rating data was used. The findings that are looming in these studies should be considered to obtain more aesthetic GUIs in order and attract the attention of users.

#### Minimalist (4.26)

The Minimalist heuristic obtained the highest average usability score in Health Companion. In fact, this PHR features a minimalist web design. Some aspects to highlight about the GUI are the existence of a simple colour scheme, without degraded colours, with the use of blue, orange, white, and grey. This set of colours generates an appropriate contrast, which facilitates the extraction of information on the web portal, and is important for its minimalist design. The colouring of web portals is fundamental in order to capture the attention of users. However, there are not many studies in literature that analyse the design of web pages that guarantee success [93]. A framework has been proposed in literature that allows for the generation of automatic colouring of web pages based on several design objectives, which are: appropriate visual contrasts, multicolour compatibility, and semantic associations. For this purpose, probabilistic data-driven models were used. The results confirmed that the system-generated designs were preferable to those generated by laypersons [94]. Another noteworthy aspect of Health Companion’s minimalist design is that it offers a GUI that is not overloaded. The use of the PHR may be influenced by this feature. It was analysed in the literature whether participants of a survey responded differently depending on how the questions were displayed on the web page. In particular, all questions were shown on a single web page in one case, and only one question on each page in another case. Most participants preferred the one-item-per-page format, even though this format was more time-consuming [95]. Health Companion is in line with this design alternative, boosting the Minimalist heuristic.

#### Consistency (4.25)

Health Companion uses strong colour contrasts to visually separate sections of the web portal. This is one of the features highlighted by the Consistency heuristic. On the welcome screen, white and dark blue shades are primarily displayed. These separate clickable objects and text boxes. In addition, animations when the cursor is located over clickable widgets can be observed. These animations range from gradual transitions to another colour, enlargement of the widget size, or underlining of words in the same way as in hyperlinks. These aspects considered in the aesthetic design of the GUI impact end users on the continuity in the use of an application [96].

#### Control (3.82)

Data entered in Health Companion can be deleted at any time. Only one exception was detected in the Activity Log section. Health Companion allows one to send e-mails with a client embedded in the system. Files containing health information can be attached to these e-mails. In addition, e-mails can only be sent to a collection of medical doctors stored in the PHR by the user. They can consult these files and thus have additional information when making diagnoses. The Activity Log is a reference that contains the timestamp of the sent e-mails. Preventing its deletion ensures that the health information provided to doctors has not been corrupted.

Widespread adoption of PHRs is hampered by technological factors such as security and privacy, between others [97]. Different mechanisms have been proposed in order to strengthen security and privacy in PHRs. As an example, an access control based on Lagrange interpolation was proposed to guarantee the security and confidentiality of health records. This mechanism allows the random generation of private keys between which there is no relation, making it difficult to crack the system [98]. New encryption schemes have also been proposed for distributed storage of clinical data, preventing the system from being vulnerable to single-point attacks [99]. An important gap has been detected in the lack of the definition of architecture standards for interfaces and data models to ensure privacy and security [100]. In this regard, a combined encryption and decryption architecture has been proposed, with AES and RSA encryption mechanisms, to encrypt medical records. The result is a more robust decentralised electronic medical record (EMR) repository [101].

#### Flexibility (3.83)

Shortcuts to the main functionalities of the system can be found in the Health Companion user profile. This feature reinforces the concept proposed in the Flexibility heuristic. The presence of shortcuts saves time for users [102]. However, alternatives to shortcuts have been proposed to make it even faster to complete the tasks. In the literature, it has been hypothesised to use sketches to learn the usage intentions in the systems, avoiding the user to access nested menus or memorise keyboard shortcuts. For this purpose, software has been used that allows the user to draw sketches on the GUI [103]. Another alternative is Guided Finger-Aware Shortcuts which aims to reduce the gap between graphical input and the activation of a shortcut. When a specific hand posture is used to press a key, this interaction approach detects it and allows complementary finger movements to select between related shortcuts if desired [104]. These options could be valued in the future when determining the HCI of systems.

## 5. Conclusions

This paper has proposed a new workflow particularly designed for usability assessments. Moreover, it has analysed the outcome of applying a methodology in which decentralisation is encouraged. Decentralisation is gaining relevance in today’s world for the promotion of access and protection of health data [105]. New decentralised technological approaches can involve a greater number of participants in audit processes, generating more extensive and detailed results [106].

The auditor’s satisfaction in using the CAUE Usevalia tool was evaluated. According to TAM, the variables evaluated have a great influence on the final use of the system [107], in this case, the application of Usevalia to perform usability audits based on heuristic inspection. The results obtained have been positive, so the evaluation of the use of Usevalia by users showed satisfactory results. By using TAM, the relationship between three variables was investigated: PEU, PA and PU. Most of the causal relationships between the variables are well supported, and the study reaffirms the appropriateness of applying TAM to measure user acceptance of the technology.

The system was found to be directly influenced by PEU, PA, and PU. The results illustrate that PEU is a key element [34] linking the variables PUand PA. This suggests that it is important to foster the user’s self-confidence that the system is easy to use. Technology is advancing rapidly and society needs time to adapt to the changes brought about by the arrival of new systems. If users find it difficult to use a system, they may genuinely believe that the system is too difficult to use and that the benefits they will gain are not worth the effort.

The usability results were analysed. The Match heuristic scored the highest, with a mean value of 3.92. This result comes from all the usability evaluations conducted in this study. This heuristic promotes that there are no major differences in terminology and the way information is presented between the real and virtual world. This finding suggests that the development of PHRs has so far focused on this approach, reducing the cognitive load [108]. This is relevant, as PHRs are intended for a wide audience, and their use should be as simple as possible [97].

A virtual environment as an alternative to the real world has great advantages for the healthcare sector [109]. Mechanisms need to be found to extend the use of PHRs. Between the alternatives, enhancing the automatic uploading of medical data from the facilities could a priori generate a wider audience. This idea is in line with the Lazy User Model [110] on the study of how actors make decisions in IS to perform the tasks. Uploading huge amounts of medical information can become an overwhelming task for users, and as a result, abandon the PHR. However, simply reviewing and querying medical data by patients would generate comfort. Several PHRs addressed this option. In NoMoreClipBoard, the feature cc:me was found, which allows the medical specialist to automatically send the information to the patient’s profile. In HealthVet, the data is displayed for premium users. It is worth noting that such practices may create responsibilities for health data. In this vein, ensuring privacy and security is crucial. Fervent debates have been generated in the academic community about the appropriateness of giving users full power over their health data [111,112].

The sense of work overload when uploading medical data to the PHR was attemped to be alleviated and motivated by offering socialisation alternatives, which improve coping conditions among patients [113]. PatientsLikeMe focuses on the concept of social networking. Users must at least indicate their conditions in order to contact other users who also have the same diseases. By receiving support from other patients, they benefit from the use of the PHR, which is the main objective. On the HealthVet portal, they promote the concept of community among ex-members in the US military. Although the PHR is not a means of communication with other users, a multitude of actions are presented to connect users in the real world with the My community section. In NoMoreClipBoard and Health Companion, no options for socialisation between users were found, although Heath Companion has recently launched the mobile application, which allows video conferencing with medical doctors. This feature of socialisation between users in the PHRs could be related to their success. The popularity of web portals was observed in the Alexa™ ranking. The results showed that those PHRs with user-to-user socialisation options ranked higher, with HealthVet ranking 2907th in popularity, PatientsLikeMe 256,628th, NoMoreClipBoard 1,598,077th and Health Companion 2,463,289th.

As future work, we propose to further develop the Usevalia tool in order to provide it with more functionality. To this end, technical features will be added to allow usability evaluations based on other methods that do not focus solely and exclusively on expert review. In addition, the possibility of extending usability evaluations to other areas such as banking, e-commerce or digital newspapers, where advertising can be very intrusive, and there is a need for mass-use web portals to be studied from a usability perspective.

Automatic testing of GUI designs could be employed to detect inconsistency issues. Widget detection in GUIs has been identified as one of the biggest challenges in automatic GUI testing [114]. This type of test is important, as they allow gathering information about triggering sequences of GUI failures. This type of test detects unnecessary events, which can be eliminated. Research results showed algorithms that obtained an effective detection rate of more than 95.4% of irrelevant events in failure sequences, by using a processing time of about 10 s [115]. Therefore, these tools should be analysed to produce GUIs more attractive to users.

## Figures and Tables

**Table 1:** Proposed checklist to guide usability evaluation.

Heuristic	Mapping Questions
H1. Match	Is the way in which the terminology of PHRs is presented familiar?
	Do the screens displayed in the PHR follow a logical order?
H2. Visibility	Are clickable widgets visually highlighted?
	Is it noticeable that the system takes excessive time to respond?
H3. Memory	Is there detailed information on how to perform the tasks?
	Can default options be found when choosing in the data menus?
H4. Minimalist	Is only the necessary information displayed?
	Is it possible to decide whether to show the information or not?
H5. Error	Does the system check user interaction to warn of possible failure?
	Do messages appear warning about the severity of user actions?
H6. Consistency	Is there consistency in the naming of different widgets that do the same thing?
	Is the grammatical style used and the terminology of the menus consistent?
H7. Control	Does the system allows to cancel a task action?
	Can I change the actions performed by users?
H8. Flexibility	Is there a possibility to use shortcuts when tasks have repetitive actions?
	Does the system offer templates that can be used in case of unnecessary actions?

**Table 2:** The collection of the tasks performed in the usability evaluation.

TASK 01: System Registration
TASK 02: System login
TASK 03: Create profile
TASK 04: Access profile
TASK 05: Access to 3rd parties management
TASK 06: Managing the medical history profile of family members
TASK 07: Managing the patient’s drugs
TASK 08: Allergies management
TASK 09: Vaccines management
TASK 10: Diseases management
TASK 11: Check current drugs
TASK 12: Generate a report
TASK 13: Check the evolution of glucose
TASK 14: Encyclopaedia of conditions
TASK 15: Export a collection of medical data
TASK 16: Schedule reminders for medical appointments and medication
TASK 17: Contact customer service or send a suggestion
TASK 18: Access to privacy policy
TASK 19: Log out
TASK 20: Password recovery

**Table 3:** TAM questionnaire used for the evaluation of Usevalia.

ID	Statements
Block 1: Perceived Usefulness (PU)	
Q01	Using Usevalia would improve my performance on the HCI course
Q02	Usevalia would favour the teaching-learning process of the HCI subject
Q03	I consider that Usevalia represents a step forward in conducting usability audits.
Q04	I would conduct future usability audits using Usevalia
Q05	The tool manages users and user roles sufficiently well to be used in a real project
Q06	The web categories seem to be sufficient to represent all the websites/applications
Q07	The different types of predefined tasks associated with the web categories seem sufficient to represent all the websites/applications
Q08	The different types of evaluation (Basic, Standard, Task Based) in the tool seem sufficient to evaluate an audit
Q09	Usevalia streamlines the audit evaluation process
Q10	The reports generated by the tool help me understand the end result of an audit
Q11	I find Usevalia’s graphs useful, as they provide interesting data on the audit
Q12	Usevalia is better than Microsoft Excel™ for performing usability audits, specifically audits with a heuristic evaluation
Block 2: Perceived Ease of Use (PEU)	
Q13	Learning to use Usevalia would be easy for me
Q14	I consider that my interaction with Usevalia would be clear and understandable
Q15	The steps to follow in the tool before creating an audit are intuitive
Q16	In general, I have no problems using and understanding the operation of Usevalia
Block 3: Perceived Attitude (PA)	
Q17	I would use Usevalia if I needed to perform usability audits
Q18	I would use Usevalia to improve my performance on the HCI course

**Table 4:** Matrix of correlations between the variables of TAM.

	PU	PEU	PA
PU	1	0.637 **	0.853 **
PEU		1	0.546 **
PA			1

** Correlation is significant at the 0.01 level (2-tailed).

**Table 5:** Average usability assessments in HealthVet.

HealthVet								
	A	B	C	D	E	F	G	Average
H1. Match	3.10	3.00	4.78	3.85	3.95	5.00	4.05	*3.96*
H2. Visibility	2.75	2.70	4.56	3.70	4.35	1.72	3.75	3.36
H3. Memory	3.20	2.85	4.28	3.55	4.45	3.61	3.75	*3.67*
H4. Minimalist	2.85	2.70	4.72	3.05	3.85	2.50	4.20	3.41
H5. Error	2.55	3.05	4.39	2.70	3.95	2.94	1.70	3.04
H6. Consistency	3.30	2.70	4.17	4.25	3.90	4.78	4.10	*3.88*
H7. Control	2.90	3.00	4.89	2.70	4.45	4.61	4.15	*3.81*
H8. Flexibility	2.25	2.70	4.78	1.10	3.95	4.61	4.30	3.38

**Table 6:** Average usability assessments in NoMoreClipBoard.

NoMoreClipBoard					
	H	I	J	K	Average
H1. Match	4.35	5.00	2.65	3.65	*3.91*
H2. Visibility	3.18	5.00	2.15	3.75	*3.52*
H3. Memory	4.24	5.00	1.80	3.35	*3.60*
H4. Minimalist	2.71	4.71	2.75	3.10	3.32
H5. Error	3.82	3.53	0.90	1.80	2.51
H6. Consistency	4.18	4.82	2.15	3.25	*3.60*
H7. Control	3.71	4.88	1.70	2.20	3.12
H8. Flexibility	4.06	5.00	1.00	2.35	3.10

**Table 7:** Average usability assessments in PatientsLikeMe (1 of 2).

PatientsLikeMe								
	L	M	N	O	P	Q	R	S
H1. Match	3.00	4.29	4.29	2.55	2.90	4.79	3.50	4.29
H2. Visibility	2.55	4.43	3.29	2.55	2.80	5.00	3.75	5.00
H3. Memory	2.10	4.50	3.93	2.85	2.50	4.50	3.75	4.93
H4. Minimalist	2.60	4.36	3.79	2.55	2.65	4.07	3.50	4.29
H5. Error	2.50	4.08	3.64	2.70	2.40	4.31	3.40	4.43
H6. Consistency	2.75	3.69	3.79	2.85	2.95	4.79	3.80	4.43
H7. Control	2.20	3.86	3.57	2.95	2.45	4.50	3.05	4.86
H8. Flexibility	2.70	4.15	3.64	2.45	2.45	4.50	3.40	4.29

**Table 8:** Average usability assessments in PatientsLikeMe (2 of 2).

PatientsLikeMe								
	T	U	V	W	X	Y	Z	Average
H1. Match	2.45	3.43	4.73	2.25	4.29	5.00	2.75	*3.63*
H2. Visibility	2.10	5.00	3.91	1.95	3.50	4.79	2.60	*3.55*
H3. Memory	1.95	3.43	4.09	2.20	3.36	4.86	2.55	3.43
H4. Minimalist	2.30	4.86	4.27	2.15	4.29	4.86	2.20	*3.51*
H5. Error	2.95	3.57	4.27	2.00	3.64	4.86	2.00	3.38
H6. Consistency	2.20	4.00	3.27	1.65	4.64	4.64	2.85	3.49
H7. Control	2.65	3.71	3.18	2.00	2.86	4.57	2.25	3.24
H8. Flexibility	1.45	3.07	3.09	0.00	2.86	4.29	2.05	2.96

**Table 9:** Average usability assessments in Health Companion.

Health Companion						
	AA	AB	AC	AD	AE	Average
H1. Match	4.50	4.50	4.00	3.25	4.59	*4.17*
H2. Visibility	3.70	3.06	2.61	3.50	4.06	3.39
H3. Memory	4.25	4.00	4.44	3.45	4.82	*4.19*
H4. Minimalist	4.45	4.39	4.78	3.35	4.35	*4.26*
H5. Error	4.50	0.89	4.39	3.00	4.00	3.36
H6. Consistency	4.25	4.50	4.78	3.20	4.53	*4.25*
H7. Control	4.20	2.22	4.61	3.30	4.76	*3.82*
H8. Flexibility	4.35	3.00	4.44	3.35	4.00	*3.83*

## Data Availability

The data presented in this study are available in http://umubox.um.es/index.php/s/IDWChOi6jy6uAyS (accessed on 27 May 2022).

## Abbreviations

The following abbreviations are used in this manuscript:

ACM	Association for Computing Machinery
AES	Advanced Encryption Standard
AHIMA	American Health Information Management Association
CAUE	Computer-Assisted Usability Evaluation
CSV	Comma Separated Values
EC	Exclusion Criteria
EMR	Electronic Medical Record
GDP	Gross Domestic Product
GUI	Graphical User Interface
HCI	Human-Computer Interaction
IC	Inclusion Criteria
ID	Identification
IEEE	Institute of Electrical and Electronics Engineers
IT	Information Technology
IS	Information System
MEM	Method Evaluation Model
PA	Perceived Attitude
PC	Personal Computer
PDF	Portable Document Format
PEU	Perceived Ease of Use
PHR	Personal Health Record
PRISMA	Preferred Reporting Items for Systematic reviews and Meta-Analysis
PU	Perceived Usefulness
RSA	Rivest, Shamir, Adleman
SMS	Short Message Service
TAM	Technology Acceptance Model
TRA	Theory of Reasoned Action
TTY	Teletypewriter
US	United States
VA	Veterans Affairs

## References

[1] Wan B., Xu C. Application of Computer Information Technology in Internet. Proceedings of the 2021 4th International Conference on Information Systems and Computer Aided Education.

[2] Yerina A., Honchar I., Zaiets S. (2021). Statistical indicators of cybersecurity development in the context of digital transformation of economy and society. Sci. Innov..

[3] Kozludzhova K. (2019). Key success factors of innovations in the software industry. Int. J. Innov. Technol. Explor. Eng..

[4] Daim T., Bukhari E., Bakry D., VanHuis J., Yalcin H., Wang X. (2021). Forecasting Technology Trends through the Gap Between Science and Technology: The Case of Software as an E-Commerce Service. Foresight.

[5] Morciano C., Errico M.C., Faralli C., Minghetti L. (2020). An analysis of the strategic plan development processes of major public organisations funding health research in nine high-income countries worldwide. Health Res. Policy Syst..

[6] Gavurova B., Kocisova K., Sopko J. (2021). Health system efficiency in OECD countries: Dynamic network DEA approach. Health Econ. Rev..

[7] Grande D., Mitra N., Iyengar R., Merchant R.M., Asch D.A., Sharma M., Cannuscio C.C. (2022). Consumer Willingness to Share Personal Digital Information for Health-Related Uses. JAMA Netw. Open.

[8] Yi Y., Zhang Z., Yang L.T., Wang X., Gan C. (2022). Edge-aided control dynamics for information diffusion in social Internet of Things. Neurocomputing.

[9] Lachance C.R., Erby L.A., Ford B.M., Allen V.C., Kaphingst K.A. (2010). Informational content, literacy demands, and usability of websites offering health-related genetic tests directly to consumers. Genet. Med..

[10] Bidargaddi N., van Kasteren Y., Musiat P., Kidd M. (2018). Developing a third-party analytics application using Australia’s National Personal Health Records System: Case study. JMIR Med. Inform..

[11] Johanssen J.O., Bernius J.P., Bruegge B. Toward usability problem identification based on user emotions derived from facial expressions. Proceedings of the 2019 IEEE/ACM 4th International Workshop on Emotion Awareness in Software Engineering (SEmotion).

[12] Percival J., McGregor C. (2016). An evaluation of understandability of patient journey models in mental health. JMIR Hum. Factors.

[13] Silveira S.A., Zaina L.A., Sampaio L.N., Verdi F.L. (2022). On the evaluation of usability design guidelines for improving network monitoring tools interfaces. J. Syst. Softw..

[14] Moody D.L. (2001). Dealing with Complexity: A Practical Method for Representing Large Entity Relationship Models. Ph.D. Thesis.

[15] Rescher N. (1979). Methodological pragmatism. Mind.

[16] Davis F.D., Bagozzi R.P., Warshaw P.R. (1989). User acceptance of computer technology: A comparison of two theoretical models. Manag. Sci..

[17] Moody D.L. (2002). Comparative evaluation of large data model representation methods: The analyst’s perspective. Proceedings of the International Conference on Conceptual Modeling.

[18] Fishbein M., Ajzen I. (1977). Belief, attitude, intention, and behavior: An introduction to theory and research. Philos. Rhetor..

[19] Hu P.J., Chau P.Y., Sheng O.R.L., Tam K.Y. (1999). Examining the technology acceptance model using physician acceptance of telemedicine technology. J. Manag. Inf. Syst..

[20] Garcia-Berna J.A., Fernandez-Aleman J.L., de Gea J.M.C., Toval A., Mancebo J., Calero C., Garcia F. (2021). Energy efficiency in software: A case study on sustainability in personal health records. J. Clean. Prod..

[21] Moher D., Liberati A., Tetzlaff J., Altman D.G., PRISMA Group (2009). Preferred reporting items for systematic reviews and meta-analyses: The PRISMA statement. Ann. Intern. Med..

[22] Nielsen J., Molich R. Heuristic evaluation of user interfaces. Proceedings of the SIGCHI Conference on Human Factors in Computing Systems.

[23] Corrao N.J., Robinson A.G., Swiernik M.A., Naeim A. (2010). Importance of testing for usability when selecting and implementing an electronic health or medical record system. J. Oncol. Pract..

[24] Zapata B.C., Fernández-Alemán J.L., Toval A., Idri A. (2018). Reusable software usability specifications for mHealth applications. J. Med. Syst..

[25] LeRouge C., Ma J., Sneha S., Tolle K. (2013). User profiles and personas in the design and development of consumer health technologies. Int. J. Med. Inform..

[26] Archer N., Fevrier-Thomas U., Lokker C., McKibbon K.A., Straus S.E. (2011). Personal health records: A scoping review. J. Am. Med. Inform. Assoc..

[27] AHIMA (2019). American Health Information Management Association. http://www.ahima.org/.

[28] Cannavò A., Calandra D., Pratticò F.G., Gatteschi V., Lamberti F. (2020). An evaluation testbed for locomotion in virtual reality. IEEE Trans. Vis. Comput. Graph..

[29] Sheppard B.H., Hartwick J., Warshaw P.R. (1988). The theory of reasoned action: A meta-analysis of past research with recommendations for modifications and future research. J. Consum. Res..

[30] Mathieson K. (1991). Predicting user intentions: Comparing the technology acceptance model with the theory of planned behavior. Inf. Syst. Res..

[31] Hariyanto D., Triyono M.B., Kohler T. (2020). Usability evaluation of personalized adaptive e-learning system using USE questionnaire. Knowl. Manag. E-Learn. Int. J..

[32] Bangor A., Kortum P., Miller J. (2009). Determining what individual SUS scores mean: Adding an adjective rating scale. J. Usability Stud..

[33] Sun Y., Wang N., Guo X., Peng Z. (2013). Understanding the acceptance of mobile health services: A comparison and integration of alternative models. J. Electron. Commer. Res..

[34] Chau P.Y., Hu P.J.H. (2001). Information technology acceptance by individual professionals: A model comparison approach. Decis. Sci..

[35] Dutta B., Peng M.H., Sun S.L. (2018). Modeling the adoption of personal health record (PHR) among individual: The effect of health-care technology self-efficacy and gender concern. Libyan J. Med..

[36] Davis F.D. (1989). Perceived usefulness, perceived ease of use, and user acceptance of information technology. MIS Q..

[37] Davis S., Wiedenbeck S. (2001). The mediating effects of intrinsic motivation, ease of use and usefulness perceptions on performance in first-time and subsequent computer users. Interact. Comput..

[38] Venkatesh V. (1999). Creation of favorable user perceptions: Exploring the role of intrinsic motivation. MIS Q..

[39] Venkatesh V., Davis F.D. (2000). A theoretical extension of the technology acceptance model: Four longitudinal field studies. Manag. Sci..

[40] Shanko G., Negash S., Bandyopadhyay T. (2016). Mobile healthcare services adoption. Int. J. Netw. Virtual Organ..

[41] Ngai E.W., Poon J., Chan Y.H. (2007). Empirical examination of the adoption of WebCT using TAM. Comput. Educ..

[42] Sánchez R.A., Hueros A.D. (2010). Motivational factors that influence the acceptance of Moodle using TAM. Comput. Hum. Behav..

[43] Ali A.X., McAweeney E., Wobbrock J.O. (2021). Anachronism by Design: Understanding Young Adults’ Perceptions of Computer Iconography. Int. J. Hum.-Comput. Stud..

[44] Sánchez A.J., Rodríguez S., de la Prieta F., González A. (2019). Adaptive interface ecosystems in smart cities control systems. Future Gener. Comput. Syst..

[45] Zhu H., Zhou Y., Wu Y., Wang X. (2022). To smile or not to smile: The role of facial expression valence on mundane and luxury products premiumness. J. Retail. Consum. Serv..

[46] Wiegand N., Imschloss M. (2021). Do You Like What You (Can’t) See? The Differential Effects of Hardware and Software Upgrades on High-Tech Product Evaluations. J. Interact. Mark..

[47] Rajesh S., Sekar A.C. (2021). Evaluation of quality attributes of software design patterns using association rules. Int. J. Adv. Intell. Paradig..

[48] Li K., Zhang J., Zhang L. (2021). Optimal Software Feature-Limited Freemium Model Design: A New Consumer Learning Theoretical Framework. Mathematics.

[49] Ruiz J., Serral E., Snoeck M. (2021). Unifying functional User Interface design principles. Int. J. Hum.-Interact..

[50] Forrin N.D., Mills C., D’Mello S.K., Risko E.F., Smilek D., Seli P. (2021). TL; DR: Longer sections of text increase rates of unintentional mind-wandering. J. Exp. Educ..

[51] Roy A., Singh A.K., Mishra S., Chinnadurai A., Mitra A., Bakshi O. (2021). Mental health implications of COVID-19 pandemic and its response in India. Int. J. Soc. Psychiatry.

[52] Hernández-Díaz Y., Genis-Mendoza A.D., Ramos-Méndez M.Á., Juárez-Rojop I.E., Tovilla-Zárate C.A., González-Castro T.B., López-Narváez M.L., Nicolini H. (2022). Mental Health Impact of the COVID-19 Pandemic on Mexican Population: A Systematic Review. Int. J. Environ. Res. Public Health.

[53] Obuobi-Donkor G., Eboreime E., Shalaby R., Agyapong B., Oluwasina F., Adu M., Owusu E., Mao W., Agyapong V.I. (2022). Evaluating the Prevalence and Predictors of Moderate to Severe Depression in Fort McMurray, Canada during the COVID-19 Pandemic. Int. J. Environ. Res. Public Health.

[54] Zaçe D., Hoxhaj I., Orfino A., Viteritti A., Janiri L., Di Pietro M. (2021). Interventions to address mental health issues in healthcare workers during infectious disease outbreaks: A systematic review. J. Psychiatr. Res..

[55] Cao Y., Ajjan H., Hong P. (2018). Post-purchase shipping and customer service experiences in online shopping and their impact on customer satisfaction: An empirical study with comparison. Asia Pac. J. Mark. Logist..

[56] Britton P.C., Karras E., Stecker T., Klein J., Crasta D., Brenner L.A., Pigeon W.R. (2022). Veterans crisis line call outcomes: Distress, suicidal ideation, and suicidal urgency. Am. J. Prev. Med..

[57] Dichter M.E., Krishnamurti L.S., Chhatre S., Hoffmire C.A., Monteith L.L., Bellamy S.L., Iverson K.M., Montgomery A.E., Agha A., McCoy I. (2022). Gender differences in veterans’ use of the Veterans Crisis Line (VCL): Findings from VCL call data. Gen. Hosp. Psychiatry.

[58] Al-Hawari F., Barham H. (2021). A machine learning based help desk system for IT service management. J. King Saud Univ.-Comput. Inf. Sci..

[59] Rawat A., Janssen M., Bargh M.S., Choenni S. Designing a user interface for improving the usability of a statistical disclosure control tool. Proceedings of the 2021 IEEE Intl Conf on Parallel & Distributed Processing with Applications, Big Data & Cloud Computing, Sustainable Computing & Communications, Social Computing & Networking (ISPA/BDCloud/SocialCom/SustainCom).

[60] Bolisani E., Scarso E., Padova A. (2018). Cognitive overload in organizational knowledge management: Case study research. Knowl. Process Manag..

[61] Habib H., Zou Y., Yao Y., Acquisti A., Cranor L., Reidenberg J., Sadeh N., Schaub F. Toggles, dollar signs, and triangles: How to (in) effectively convey privacy choices with icons and link texts. Proceedings of the 2021 CHI Conference on Human Factors in Computing Systems.

[62] Khamaj A., Kang Z., Argyle E. (2019). Users’ perceptions of smartphone weather applications’ usability. Proceedings of the Human Factors and Ergonomics Society Annual Meeting.

[63] van Dellen J.R. (2021). Chiari malformation: An unhelpful eponym. World Neurosurg..

[64] Ye J., Chen K., Xie X., Ma L., Huang R., Chen Y., Xue Y., Zhao J. An empirical study of GUI widget detection for industrial mobile games. Proceedings of the 29th ACM Joint Meeting on European Software Engineering Conference and Symposium on the Foundations of Software Engineering.

[65] Su D., Liu J., Zhu S., Wang X., Wang W., Zhang X. (2021). AppQ: Warm-starting App Recommendation Based on View Graphs. arXiv.

[66] Bunjak A., Černe M., Popovič A. (2021). Absorbed in technology but digitally overloaded: Interplay effects on gig workers’ burnout and creativity. Inf. Manag..

[67] Cao Y., Proctor R.W., Ding Y., Duffy V.G., Zhang Y., Zhang X. (2021). Influences of Color Salience and Location of Website Links on User Performance and Affective Experience with a Mobile Web Directory. Int. J. Hum.-Interact..

[68] Balajee R., Jayanthi Kannan M., Murali Mohan V. (2022). Web Design Focusing on Users Viewing Experience with Respect to Static and Dynamic Nature of Web Sites. Inventive Computation and Information Technologies.

[69] Moreno L., Valencia X., Pérez J.E., Arrue M. (2021). An exploratory study of web adaptation techniques for people with low vision. Univers. Access Inf. Soc..

[70] Meyer A.N., Upadhyay D.K., Collins C.A., Fitzpatrick M.H., Kobylinski M., Bansal A.B., Torretti D., Singh H. (2021). A program to provide clinicians with feedback on their diagnostic performance in a learning health system. Jt. Comm. J. Qual. Patient Saf..

[71] Balajee R., Mohapatra H., Deepak V., Babu D.V. Requirements identification on automated medical care with appropriate machine learning techniques. Proceedings of the 2021 6th International Conference on Inventive Computation Technologies (ICICT).

[72] Morris A.H., Stagg B., Lanspa M., Orme J., Clemmer T.P., Weaver L.K., Thomas F., Grissom C.K., Hirshberg E., East T.D. (2021). Enabling a learning healthcare system with automated computer protocols that produce replicable and personalized clinician actions. J. Am. Med. Inform. Assoc..

[73] Kunene K.N., Diop M. A lazy user perspective to the voluntary adoption of electronic personal health records (PHRs). Proceedings of the 51st Hawaii International Conference on System Sciences.

[74] Behnke M., Valik J.K., Gubbels S., Teixeira D., Kristensen B., Abbas M., van Rooden S.M., Gastmeier P., van Mourik M.S., Aspevall O. (2021). Information technology aspects of large-scale implementation of automated surveillance of healthcare-associated infections. Clin. Microbiol. Infect..

[75] Chelladurai U., Pandian S. (2022). A novel blockchain based electronic health record automation system for healthcare. J. Ambient. Intell. Humaniz. Comput..

[76] de Chantal P.L., Markovits H. (2022). Reasoning outside the box: Divergent thinking is related to logical reasoning. Cognition.

[77] Wang H., Guo B., Wu W., Liu S., Yu Z. (2021). Towards information-rich, logical dialogue systems with knowledge-enhanced neural models. Neurocomputing.

[78] Simon K., Hurst M. (2021). Body Positivity, but not for everyone: The role of model size in exposure effects on women’s mood, body satisfaction, and food choice. Body Image.

[79] Ross K., VanNortwick M. (2021). Managing mood-related symptoms utilizing diet, targeted nutrient supplementation, and lifestyle changes: A case series. EXPLORE.

[80] Dhensa-Kahlon R.K., Woods S.A. (2022). Humor styles as markers of personality facets: An examination of the personality structural foundation of humor. Personal. Individ. Differ..

[81] Yang F., Wen D. (2021). Combating workplace loneliness climate and enhancing team performance: The roles of leader humor and team bureaucratic practices. J. Bus. Res..

[82] Papadopoulos I. (2019). Using mobile puzzles to exhibit certain algebraic habits of mind and demonstrate symbol-sense in primary school students. J. Math. Behav..

[83] McKee L.G., Algoe S.B., Faro A.L., O’Leary J.L., O’Neal C.W. (2019). Picture This! Bringing joy into Focus and Developing Healthy Habits of Mind: Rationale, design, and implementation of a randomized control trial for young adults. Contemp. Clin. Trials Commun..

[84] Lee Y.J., Kim M.A., Park H.J. (2020). Effects of a laughter programme with entrainment music on stress, depression, and health-related quality of life among gynaecological cancer patients. Complement. Ther. Clin. Pract..

[85] Altmann B.A., Gertheiss J., Tomasevic I., Engelkes C., Glaesener T., Meyer J., Schäfer A., Wiesen R., Mörlein D. (2022). Human perception of color differences using computer vision system measurements of raw pork loin. Meat Sci..

[86] Niu Y., Liu J., Cui J., Yang W., Zuo H., He J., Xiao L., Wang J., Ma G., Han Z. (2022). Research on visual representation of icon colour in eye-controlled systems. Adv. Eng. Inform..

[87] Shen Z., Zhang L., Li R., Hou J., Liu C., Hu W. (2021). The effects of color combinations, luminance contrast, and area ratio on icon visual search performance. Displays.

[88] Mu D., Huang Y., Wang Y., Yang J., Li J., Kang Z. (2022). Neumorphic or flat? Impact of icon depth on user attention and visual search efficiency. Int. J. Ind. Ergon..

[89] Kopp T., Riekert M., Utz S. (2018). When cognitive fit outweighs cognitive load: Redundant data labels in charts increase accuracy and speed of information extraction. Comput. Hum. Behav..

[90] Deng L., Poole M.S. (2012). Aesthetic design of e-commerce web pages–Webpage Complexity, Order and preference. Electron. Commer. Res. Appl..

[91] Wan H., Ji W., Wu G., Jia X., Zhan X., Yuan M., Wang R. (2021). A novel webpage layout aesthetic evaluation model for quantifying webpage layout design. Inf. Sci..

[92] Dou Q., Zheng X.S., Sun T., Heng P.A. (2019). Webthetics: Quantifying webpage aesthetics with deep learning. Int. J. Hum.-Comput. Stud..

[93] Lee Y., Coyle J.R., Chen A.N. (2021). Improving intention to back projects with effective designs of progress presentation in crowdfunding campaign sites. Decis. Support Syst..

[94] Gu Z., Lou J. (2016). Data driven webpage color design. Comput.-Aided Des..

[95] Thorndike F.P., Carlbring P., Smyth F.L., Magee J.C., Gonder-Frederick L., Ost L.G., Ritterband L.M. (2009). Web-based measurement: Effect of completing single or multiple items per webpage. Comput. Hum. Behav..

[96] Bessghaier N., Soui M., Ghaibi N. (2022). Towards the automatic restructuring of structural aesthetic design of Android user interfaces. Comput. Stand. Interfaces.

[97] Harahap N.C., Handayani P.W., Hidayanto A.N. (2022). Barriers and Facilitators of Personal Health Record Adoption in Indonesia: Health Facilities’ Perspectives. Int. J. Med. Inform..

[98] Huang Y.T., Chiang D.L., Chen T.S., Wang S.D., Lai F.P., Lin Y.D. (2022). Lagrange interpolation-driven access control mechanism: Towards secure and privacy-preserving fusion of personal health records. Knowl.-Based Syst..

[99] Sarosh P., Parah S.A., Bhat G.M., Heidari A.A., Muhammad K. (2021). Secret sharing-based personal health records management for the internet of health things. Sustain. Cities Soc..

[100] Keshta I. (2022). AI-driven IoT for smart health care: Security and privacy issues. Inform. Med. Unlocked.

[101] Lee Y.L., Lee H.A., Hsu C.Y., Kung H.H., Chiu H.W. (2022). SEMRES-A triple security protected blockchain based medical record exchange structure. Comput. Methods Programs Biomed..

[102] Mooers B.H. (2020). Shortcuts for faster image creation in PyMOL. Protein Sci..

[103] Samuelsson S.G., Book M. Towards Sketch-based User Interaction with Integrated Software Development Environments. Proceedings of the IEEE/ACM 42nd International Conference on Software Engineering Workshops.

[104] Zheng J., Lewis B., Avery J., Vogel D. Fingerarc and fingerchord: Supporting novice to expert transitions with guided finger-aware shortcuts. Proceedings of the 31st Annual ACM Symposium on User Interface Software and Technology.

[105] Mondal S., Shafi M., Gupta S., Gupta S.K. (2022). Blockchain Based Secure Architecture for Electronic Healthcare Record Management. GMSARN Int. J..

[106] Yuan J., Njilla L. Lightweight and Reliable Decentralized Reward System using Blockchain. Proceedings of the IEEE INFOCOM 2021-IEEE Conference on Computer Communications Workshops (INFOCOM WKSHPS).

[107] Al-Adwan A., Al-Adwan A., Smedley J. (2013). Exploring students acceptance of e-learning using Technology Acceptance Model in Jordanian universities. Int. J. Educ. Dev. Using ICT.

[108] Saribatur Z.G., Eiter T., Schüller P. (2021). Abstraction for non-ground answer set programs. Artif. Intell..

[109] Trenfield S.J., Awad A., McCoubrey L.E., Elbadawi M., Goyanes A., Gaisford S., Basit A.W. (2022). Advancing pharmacy and healthcare with virtual digital technologies. Adv. Drug Deliv. Rev..

[110] Collan M., Tétard F. Lazy user theory of solution selection. Proceedings of the IADIS International Conference Cognition and Exploratory Learning in Digital Age 2007.

[111] Karatas M., Eriskin L., Deveci M., Pamucar D., Garg H. (2022). Big Data for Healthcare Industry 4.0: Applications, challenges and future perspectives. Expert Syst. Appl..

[112] Annane B., Alti A., Lakehal A. (2022). Blockchain based context-aware CP-ABE schema for Internet of Medical Things security. Array.

[113] Faro A.L., McKee L.G., Garcia R.L., O’Leary J.L. (2019). Emotion socialization, social connectedness and internalizing symptoms in emerging adults. J. Appl. Dev. Psychol..

[114] Nass M., Alégroth E., Feldt R. (2021). Why many challenges with GUI test automation (will) remain. Inf. Softw. Technol..

[115] Yan J., Zhou H., Deng X., Wang P., Yan R., Yan J., Zhang J. (2021). Efficient testing of GUI applications by event sequence reduction. Sci. Comput. Program..

